# Optimization of a Single Tube Practical Acoustic Thermometer

**DOI:** 10.3390/s20051529

**Published:** 2020-03-10

**Authors:** Rok Tavčar, Janko Drnovšek, Jovan Bojkovski, Samo Beguš

**Affiliations:** Laboratory of Metrology and Quality, Faculty of Electrical Engineering, University of Ljubljana, 1000 Ljubljana, Slovenia; janko.drnovsek@fe.uni-lj.si (J.D.); jovan.bojkovski@fe.uni-lj.si (J.B.); samo.begus@fe.uni-lj.si (S.B.)

**Keywords:** acoustic waveguide, thermometer, temperature, speed of sound, real gas, Monte Carlo simulation

## Abstract

When designing a single tube practical acoustic thermometer (PAT), certain considerations should be addressed for optimal performance. This paper is concerned with the main issues involved in building a reliable PAT. It has to be emphasised that a PAT measures the ratio of the time delay between the single temperature calibration point (ice point) and any other temperature. Here, we present different models of the speed of sound in tubes, including the effects of real gases and an error analysis of the most accurate model with a Monte Carlo simulation. Additionally, we introduce the problem of acoustic signal overlap and some possible solutions, one of which is acoustic signal cancellation, which aims to eliminate the unwanted parts of an acoustic signal, and another is to optimize the tube length for the parameters of the gas used and specific temperature range.

## 1. Introduction

Temperature measurements are important for advancements in science [1] and industrial processes [2,3], often requiring specialised thermometers [4] of various designs [5]. Metrologically, instruments are divided into two groups: primary and secondary instruments. Primary instruments measure a physical quantity using the indirect measurement of other physical quantities along with physical constants. In contrast, secondary instruments measure the physical quantity relative to a known quantity. Primary thermometers cannot always be used as they are not well suited for some industrial environmental parameters, such as humidity, electromagnetic (EM) noise, or vibration. Primary thermometers include classical gas thermometers, noise thermometers, acoustic gas thermometers, total radiation thermometers, dielectric constant gas thermometers, and Johnson noise thermometers, etc.

The acoustic gas thermometer (AGT) is the most accurate primary thermometer and is mainly used to determine the Boltzmann constant [1]. One of the drawbacks of AGT is that current designs require laboratory conditions and specialised laboratory equipment for operation. Recent adaptations of an AGT have adopted more practical designs for use in less controlled environments [6,7], i.e., the so-called practical acoustic thermometer (PAT). The PAT differs from the AGT in that PATs are not primary thermometers because their design does not permit the measurement of all necessary quantities: however, they are more practical to make and use. For single- and multi-tube PATs, the length of the sensing tube must be determined through calibration. The main advantage of PATs over currently used secondary thermometers is the reduced need for calibration points over a given measuring range. For example, platinum resistance thermometers with a temperature range of −100 °C to 300 °C would need seven measurements, i.e., calibration points. The goal is to create an accurate instrument (and corresponding model) that only needs one calibration point for the whole temperature range. Most new designs for PATs have changed the physical geometry of the AGT to reduce its size. These adaptations focused on researching easier and more precise ways to measure the speed of sound in a medium at a given sound path geometry.

**Figure 1:**
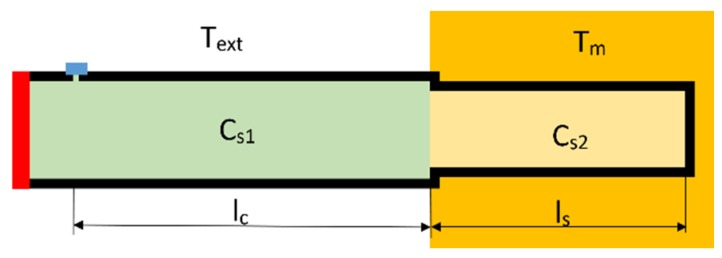
Schematic diagram of single tube practical acoustic thermometer (PAT). The position of the loudspeaker is marked in red and the position of the microphone is marked in blue.

The early PAT designs used a resonant cavity [8] and a single tube with two different radii [9], whereas newer designs [10,11] use twin tubes of different lengths. The difficulty with the resonant cavity design is that it is still not practical compared with other types of secondary thermometer. The problem with the multi tube design is maintaining an equal temperature in both tubes. The single tube design consists of one tube with a change of diameter, as shown in Figure 1, where *c_s1_* and *c_s2_* are the speed of sound inside the tube, *T_ext_* is the environmental temperature, *T_m_* is the measured temperature, *l_c_* is the length of the common sound path, and *l_s_* is length of the sensing end of the thermometer. The difference between single and multi-tube designs is that the single tube design uses one tube with a change in radius, and the multi tube design uses two physically separate tubes. Compared with the multi tube design, the single tube has the advantage of having the same temperature in all common sound paths (as there is only one path) and it only requires one microphone. However, it has the disadvantage of higher acoustic attenuation, due to soundwave reflection, and of acoustic signal overlapping. Acoustic overlap happens when two or more acoustic signals arrive to a microphone at same time.

In this study, two ideas are presented for solving the acoustic signal overlap problem in a single tube PAT. The first solution is to eliminate part of the returned acoustic signal and the second is to find the optimal tube lengths. Common to all PAT designs is the relationship between temperature and the speed of sound. So far, only theoretical or simplified models have been considered. To improve such models, it is necessary to consider the effects of real gases, such as the second acoustic virial coefficient [12]. For the design of acoustic thermometers similar to that of this study, it is also important to model the pressure, composition and mixing of the measurement gas inside the PAT [13,14] after the thermometer is sealed as this additionally effects dynamic parameters of the thermometer [15]. The gas pressure inside the thermometer also influences the attenuation of sound waves inside the tubes and the acoustic signal-to-noise ratio.

Finally, we tested a novel idea for measuring the speed of sound in tubes by using a frequency modulated continuous wave acoustic signal.

## 2. Relation between Temperature and the Speed of Sound

Calculating temperature from the speed of sound in a tube can be done with varying accuracy and complexity [16,17,18,19]. Previous attempts have assumed that the speed of sound in the tubes is the same as in a free field or they have used simplified versions of complex equations governing the transmission of sound in tubes. While the simplified versions were a great improvement over the free field speed of sound, some of the assumptions made while deriving the simplified versions were violated by the acoustic thermometer design (e.g., the inner radius of tube has to be much smaller than the wavelength of sound waves). To test the validity of simplified equations for the design, it is necessary to compare the results with a complete solution to the problem of sound travelling in cylindrical tubes. Also, in real gas the speed of sound is dependent on pressure as opposed to an ideal gas. This effect is modelled by a second acoustic virial coefficient in the equation for the free field speed of sound.

### 2.1. Free Field Model of the Speed of Sound

The simplest model of the speed of sound in tubes is the free field speed of sound, which disregards all effects of tubes on the speed of sound. The effects of real gases are modelled using a second acoustic virial coefficient. The free field speed of sound can be calculated from:

(1) $$c_{f}=\sqrt{\frac{\gamma RT}{M}}\left(1+\beta_{a}\left(T\right)\rho \right),$$

where γ is the ratio of specific heat values of ideal gas, *M* is molar mass, R is universal gas constant, *T* is temperature in kelvin, *β_a_* is second acoustic virial coefficient, and *ρ* is density.

### 2.2. Complete Model of the Speed of Sound in Tubes

To calculate temperature from the speed of sound in cylindrical tubes [18], one needs to know the free field speed of sound in the medium inside the tubes and the propagation constant of sound:

(2) $$c_{s}=\frac{c_{f}}{Im\left[\Gamma \;\right]},$$

where *Im*[*Γ*] is the imaginary part of the propagation constant of the first propagation mode for sound in tubes. The propagation constant *Γ* for cylindrical tubes with constant circular cross-section can be calculated from (*Y* = *Γ*^2^):

(3) $$Y{\left(Y-i\frac{s^{2}}{k^{2}}\right)}^{-\frac{1}{2}}\left(\frac{1}{x_{1}}-\frac{1}{x_{2}}\right)\frac{J_{1}\left(\alpha_{1}\right)}{J_{0}\left(\alpha_{1}\;\right)}+\left(\frac{\gamma k^{2}}{\sigma^{2}s^{2}}-\frac{i}{x_{1}}\right){\left(Y-x_{1}\right)}^{\frac{1}{2}}\frac{J_{1}\left(\alpha_{2}\right)}{J_{0}\left(\alpha_{2}\right)}-\;\left(\frac{\gamma k^{2}}{\sigma^{2}s^{2}}-\frac{i}{x_{2}}\right){\left(Y-x_{2}\right)}^{\frac{1}{2}}\frac{J_{1}\left(\alpha_{3}\right)}{J_{0}\left(\alpha_{3}\right)}\;=0$$

where σ is the square root of the Prandtl number, *i* is imaginary unit, *Jn* is Bessel function of first kind of order *n*, and *s* and *k* are:

(4) $$s=R\sqrt{\frac{\rho \omega }{\mu }\;}$$

(5) $$k=\frac{\omega R}{c_{f}},$$

where *R* is inner radius of the tube, *ω* is angular frequency of oscillation of the gas, *ρ* is density, and *µ* is dynamic viscosity. α1, α2, α3 are:

(6) $$\alpha_{1}=k{\left(Y-i\frac{s^{2}}{k^{2}}\right)}^{\frac{1}{2}}$$

(7) $$\alpha_{2}=k{\left(Y-x_{1}\right)}^{\frac{1}{2}}$$

(8) $$\alpha_{3}=k{\left(Y-x_{2}\right)}^{\frac{1}{2}}$$

and *x*_1,2_ are small and large roots of:

(9) $$1+\left(1+i\frac{k^{2}}{s^{2}}\left(\frac{4}{3}+\frac{\gamma }{\sigma^{2}}\right)\right)x+i\frac{\gamma^{2}k^{2}}{\sigma^{2}s^{2}}\left(\frac{1}{\gamma }+i\frac{4}{3}\frac{k^{2}}{s^{2}}\right)x^{2}=0$$

Calculating *Y* from Equation (3) is done iteratively, where the temperature estimated from the previous step is used to calculate gas parameters and re-calculate the temperature. Convergence is quick, requiring less than four iterations to achieve an error smaller than 1 mK. The starting value of *Y* can be a value of *Γ* from one iteration of the simplified model, which does not require knowledge of *Y* or *Γ*.

### 2.3. Simplified Model of the Speed of Sound in Tubes

For a simplified model of the propagation constant *Γ* [19] one can get:

(10) $$\Gamma =\sqrt{\frac{J_{0}\left(i^{\frac{3}{2}}S\right)}{j_{2}\left(i^{\frac{3}{2}}S\right)}}\sqrt{\gamma +\left(\gamma -1\right)\frac{J_{2}\left(i^{\frac{3}{2}}\sqrt{\sigma }S\right)}{J_{0}\left(i^{\frac{3}{2}}\sqrt{\sigma }S\right)}}$$

with the assumptions that *k* ≪ 1 and *k*/*s* ≪ 1.

Simplified Equation (10) is used, because it significantly reduces computational cost (10×) of the computation of the temperature from measured speed of sound in tubes compared to Equations (3) to (9). Because stated assumptions limit its use, it is necessary to compare results of both models with specific parameters of PAT design.

### 2.4. Comparison of Speed of Sound Models

**Figure 2:**
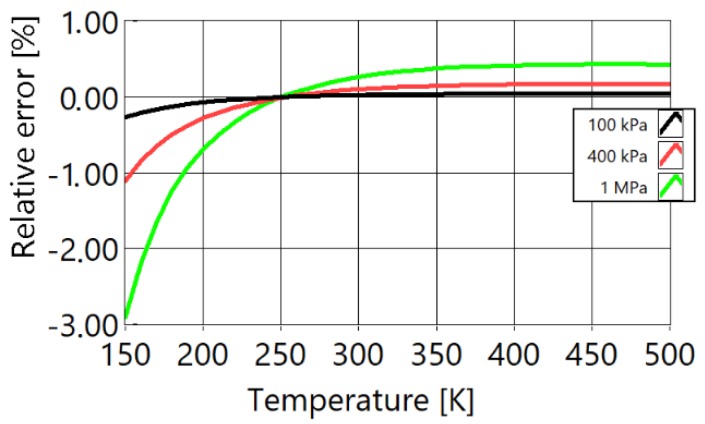
Comparison of ideal value of free field speed of sound with the full model that includes the effects of real gases. With black is marked isobar of 100 kPa, with red is marked isobar of 400 kPa and with green is marked isobar of 1 MPa of pressure inside of PAT waveguides.

Figure 2 shows the relative differences of speed of sound versus temperature for the theoretical speed of sound in a free field and speed of sound in a free field with real gas effects taken into account.

The free field ideal value of speed of sound is defined as $c_{f}=\sqrt{\gamma RT/M}$ and is represented by the line at 0%. The difference from this ideal value at 100 kPa (black), 400 kPa (red), and at 1 MPa (green) is shown. It can be seen that the ideal speed of sound in a free field at low temperatures is significantly different to the measured speed in real gases (more than 0.5% at 400 kPa and more than 2.5% at 1 MPa). To achieve a temperature error of less than 1%, it is necessary to achieve a speed of sound error of less than 0.5%, due to a quadratic relationship. For measuring low temperatures (400 kPa and −100 °C), the maximum difference in the speed of sound is more than 0.6%, but for thermometers with higher temperature ranges (600 to 1000 °C), this may be acceptable as the maximum difference is 0.3%.

**Figure 3:**
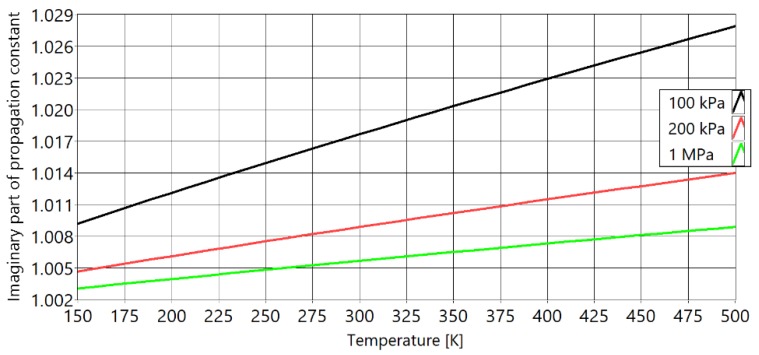
Imaginary part of the propagation constant of the full model for different values of pressure inside of PAT waveguide. With black is marked isobar of 100 kPa, with red is marked isobar of 400 kPa and with green is marked isobar of 1 MPa.

**Figure 4:**
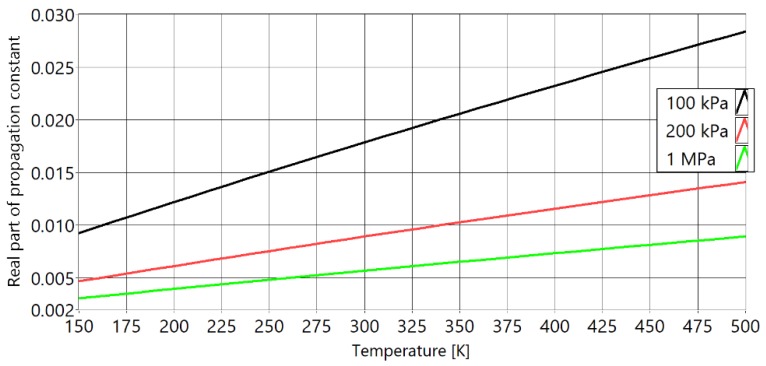
Real part of the propagation constant of the model for different values of pressure inside of PAT waveguides. With black is marked isobar of 100 kPa, with red is marked isobar of 400 kPa and with green is marked isobar of 1 MPa.

Figure 3 shows the imaginary part of the propagation constant Γ, for the complete model versus temperature at different pressures. With increasing pressure, the imaginary part of the propagation constant is converging towards 1 (free field speed of sound). Figure 4 presents the real part of the same propagation constant. The real part quantifies the attenuation of the amplitude of sound. While it is not directly related to the measurement of the temperature, it is important because it contributes significantly to the signal-to-noise ratio (SNR) of the acoustic signal. With increasing pressure, the attenuation of sound decreases.

The ratio *k*/*s* was below 0.004 for simulated pressure, tube radius, and temperature range, and therefore it satisfies the condition *k*/*s* << 1. However, ratio *k* was between 0.2 and 0.4 in this temperature range and did not satisfy the condition *k* << 1. For this reason, it was necessary to compare complete and simplified models to confirm that results of Equation (10) match results from complete model of propagation of sound waves in tubes.

**Figure 5:**
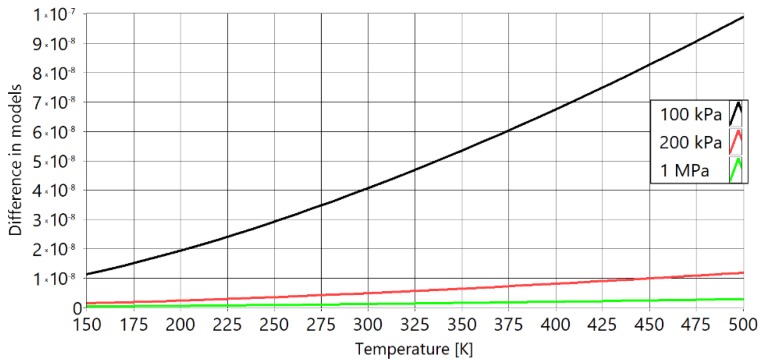
Error of the simplified model for different pressures for the imaginary part of the propagation constant *Γ*.

Figure 5 shows the differences between the imaginary part of the propagation constant for the complete and simplified models. It can be seen that, for this design and the physical constants used, the simplified model is a good approximation (maximum difference of only 1.0 × 10^−7^) despite violating one of the assumptions in deriving the simplified model. In comparison imaginary part would need to change more than 5.6 × 10^−4^ to change result of temperature calculation for 1 mK at 0 °C and 100 kPa. Similar to Figure 3 and Figure 4, the differences decrease as the pressure inside the tubes increases.

## 3. Materials and Methods

The PAT prototype is constructed from two main parts, the PAT housing and the acoustic connector. The PAT housing includes two acoustic waveguides (sensing end and common sound path) and a lid with electrical and pneumatic connections. The acoustic connector is used for mounting the microphone and the loudspeaker to the acoustic waveguide.

### 3.1. PAT Housing

**Figure 6:**
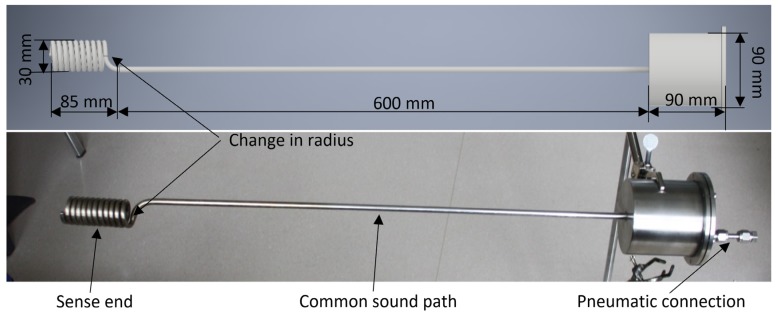
CAD model and photo of the PAT housing.

In Figure 6 there is a picture of the PAT (bottom) and the CAD model (top). On the left is the tube wound in a helix (sensing end of PAT) with an untwisted length of 0.9 m, in the middle is the common sound path, on the right is the housing for the acoustic connector and connections to outside (electrical connector is near the pneumatic connector, but is not visible in Figure 6). The inner diameter of the common sound path is 6 mm and the inner diameter of the sensing end is 4 mm. The PAT housing was made from stainless steel ALSI 304. It can withstand an internal pressure of 10 bar. This prototype was used for testing the acoustic signal cancellation algorithm. To test the algorithm with optimal lengths regarding the signal overlap, another PAT with different dimensions was designed and tested. The length of the common sound path was 0.8 m and length of the sensing end was 0.45 m. All the other dimensions were the same for both prototypes.

### 3.2. Acoustic Connector

**Figure 7:**
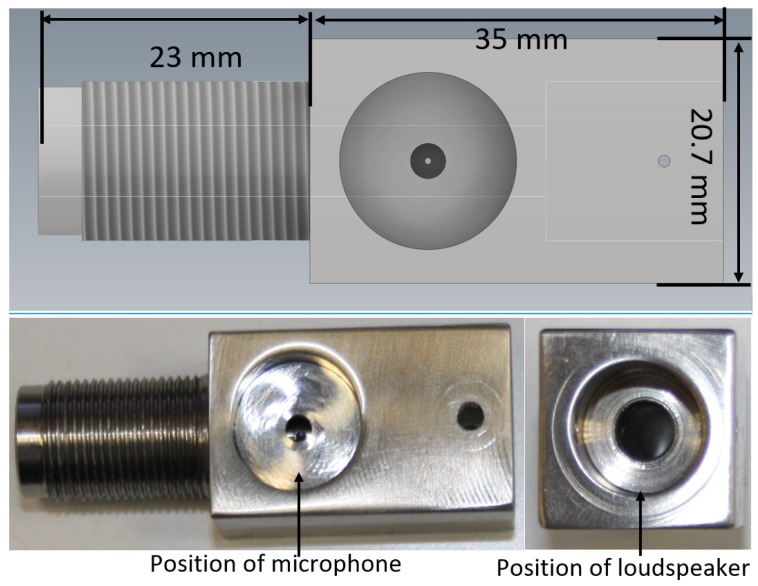
CAD model and photo of the acoustic connector.

**Figure 8:**
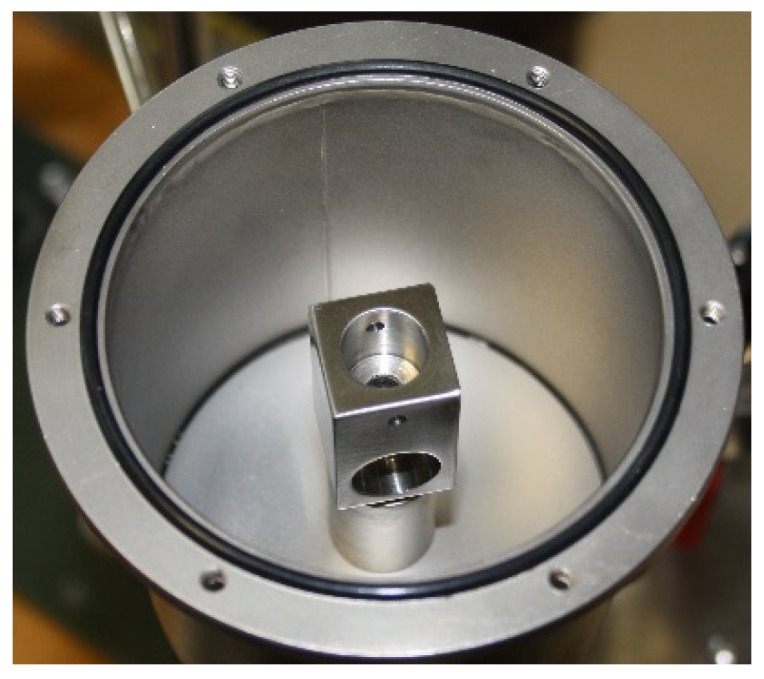
Position of the acoustic connector mounting on the PAT housing.

In Figure 7, there is a picture and the CAD model of the acoustic connector. The microphone is fitted on the centre of the acoustic connector and the loudspeaker is fitted at the right end. On the other side of the microphone there is a small hole for pressure equalisation between the acoustic waveguide and the rest of the PAT housing. Both the microphone and the loudspeaker are fitted tightly to the acoustic connector to prevent vibrations moving them. Similarly, the acoustic connector is firmly screwed to the rest of the PAT housing, as shown in Figure 8.

### 3.3. Microphone and Loudspeaker

**Figure 9:**
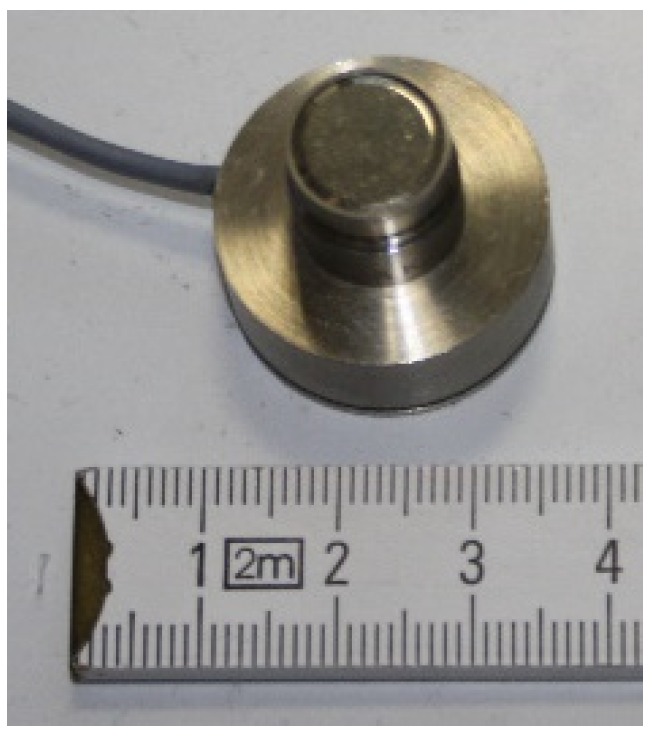
Picture of the microphone, B&K Type 4189.

The microphone that is used is a condenser pre-polarized measurement microphone B&K Type 4189 and is shown in Figure 9. Mounted on the back side of the microphone is an adapter for the electrical connection between the microphone cartridge and the microphone amplifier.

The microphone is mounted on the acoustic connector without a protection grid to reduce the air gap between the microphone membrane and the acoustic waveguide. The microphone is mounted perpendicular to the acoustic waveguide.

For the loudspeaker another B&K Type 4189 microphone was used, but in this case, it was driven with an electrical signal. It was chosen because it has a broader frequency response and less ringing than ordinary dynamic loudspeakers. The loudspeaker is mounted at end of the acoustic waveguide and parallel to the sound waves.

### 3.4. Electronic Circuits

**Figure 10:**
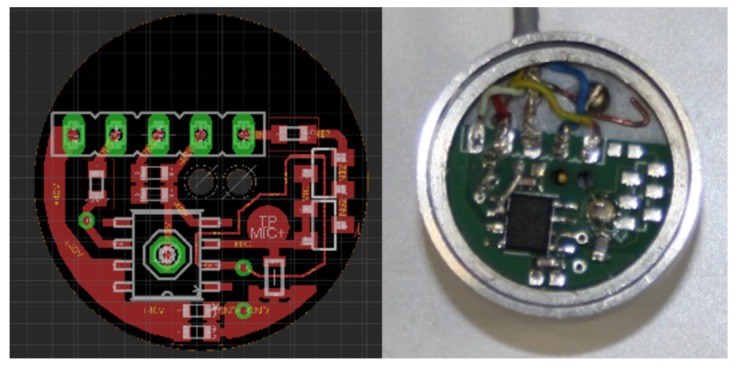
Board design (**left**) and mounting of the printed circuit board inside the Brüel & Kjær AQ-0015 adapter (**right**).

In the PAT, two electronic circuits are used, one is for microphone signal amplification and the other for loudspeaker electrical signal amplification. The microphone preamplifier is positioned behind and close to the microphone and built in the Brüel & Kjær AQ-0015 adapter, as can be seen in Figure 10.

**Figure 11:**
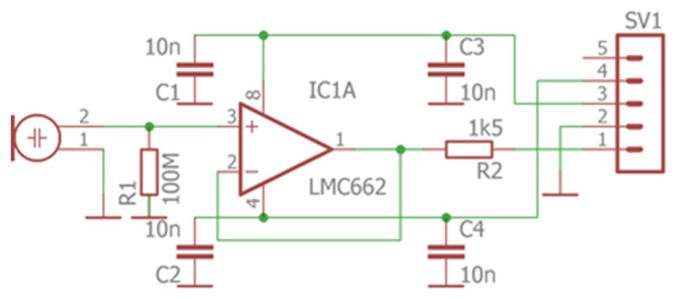
Electrical schematic of the microphone preamplifier.

In Figure 11 there is an electrical schematic of the microphone preamplifier. The scheme differs slightly from the board design in Figure 10, as the same board can be used for three slightly different designs.

Using a microphone with a long cable is problematic, as the cable can have high capacitance, which would filter the electric signal from the microphone. This is solved by positioning the PCB with an operation amplifier to buffer electric signals closer to microphone.

### 3.5. Pressure Measurement

The measurement gas used in the PAT was argon at overpressure relative to outside. The pressure inside this PAT prototype was 4 bar. The reason for the increased pressure is that previous tests [12] showed that increased pressure increases the SNR of acoustic delay measurements. The pressure was measured and regulated by a pressure controller, DPI 515 by Druck.

### 3.6. Signal Shape

Two kinds of acoustic signals are used, the first is a chirp signal to find the rough time delay and gated sine wave signal, which is used to determine the precise time delay. Chirp signal is defined as:

(11) $$y_{sweep}\left(t\right)=\{\begin{array}{c} A\sin\left(2\pi \left(\frac{f_{2}-f_{1}}{2T}t^{2}+f_{1}t\right)\right),\;0<t<t_{max}\;\\ \;\;\;\;\;\;\;\;0,\;otherwise \end{array}$$

where *A* is amplitude of signal, *f_1_* is start frequency, *f_2_* is stop frequency and *t_max_* is duration of signal. Gated sine wave signal is defined as:

(12) $$y_{sine}\left(t\right)=\{\begin{array}{c} A\;\sin\left(2\pi ft\right),\;0<t<t_{max}\\ \;\;\;0,\;otherwise \end{array}$$

where *f* is frequency. The duration of each signal was 1 ms. Start and stop frequency of chirp were 3 kHz and 7 kHz. Choices for these two frequencies are limited by duration of signal and by attenuation of sound waves. Frequency of gated sine wave was 6 kHz. Amplitude was set to utilize full range of used sound card.

**Figure 12:**
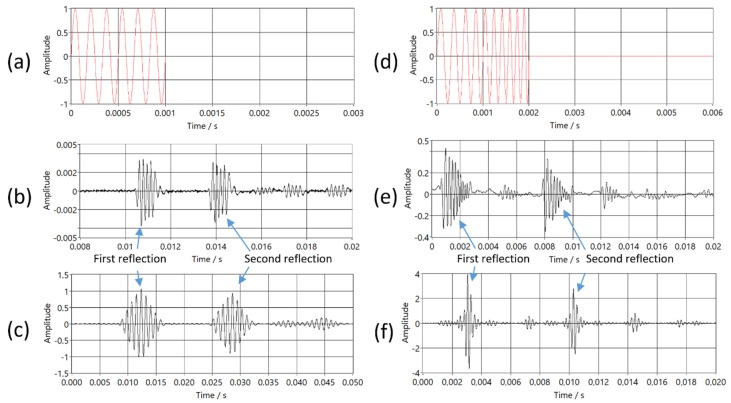
Result of the cross correlation between the transmitted and received signals for gated sinewave and chirp: (**a**) excitation signal for gated sine wave; (**b**) received signal from gated sine wave excitation; (**c**) result of cross-correlation for gated sine wave; (**d**) excitation signal for chirp signal; (**e**) received signal from chirp excitation; (**f**) result of cross-correlation for chirp.

One reason for using the chirp signal is that the cross-correlation of the gate sine signal does not have one significant peak, as is shown in Figure 12.

Chirp signals can be used alone, however the received signal from gated sine wave excitation had higher SNR in our PAT prototype, and it is easier to calculate temperature from a measured time delay of gated sine waves.

### 3.7. PAT Block Diagram

**Figure 13:**
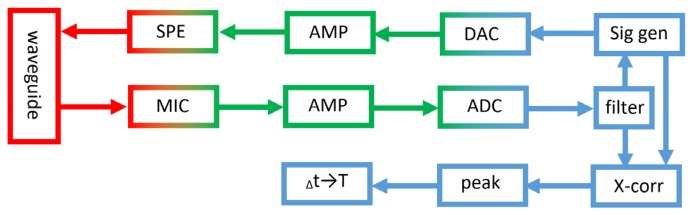
Block diagram of the PAT operations.

In Figure 13 there is a block diagram of the PAT. The digital signals are marked in blue, the analogue signals in green, and the acoustic signals in red. Generated signals are sent to the loudspeaker through a digital to analogue converter and amplifier. The loudspeaker transmits acoustic signals into the acoustic waveguide. The microphone receives signals from the acoustic waveguide and transforms them into electric signals. The signals are then amplified and digitalised. Next, a digital filter is used to remove noise outside of frequency band of interest. If acoustic signal cancellation is used, then the previous signal influences the next signal generated. The last part of the signal processing determines the time delay between acoustic signals by finding peaks in cross-correlation between the transmitted signal and the received signal. The temperature is calculated from this time delay and Equations (1) to (9), so that the modelled and measured speed of sound matches.

## 4. Uncertainty Analysis

An important part of designing a new instrument is to determine the uncertainty budget of its measurements. Uncertainty can be either calculated analytically or by simulation. Simulation is commonly used when no analytical solution is known or when it is too complicated to calculate the partial derivatives of the mathematical model of the instrument. In our case, the model for this instrument is highly complex; therefore, the uncertainty was calculated by the means of a Monte Carlo (MC) simulation of the model based on Equation (3). The MC simulation was implemented according to the “Guide to the expression of uncertainty in measurement” and its supplement 1 [20]. The uncertainty of the parameters of Equation (3) were gathered from the NIST webbook (gas properties), from the used instrument (pressure sensor) datasheets, and from the sound delay measurements. In Table 1, the parameters of the model with the associated uncertainties and probability distributions at 0 °C and a pressure of 400 kPa are presented.

All the parameters were considered to be uncorrelated, except the heat capacities *c_p_* and *c_v_* where a covariance of 1 was used. MC simulation consisted of one million repetitions. Number of repetitions was selected in order to satisfy condition given in “Guide to the expression of uncertainty in measurement” and its supplement 1 [20].

## 5. The Acoustic Signal Overlap Problem

**Figure 14:**
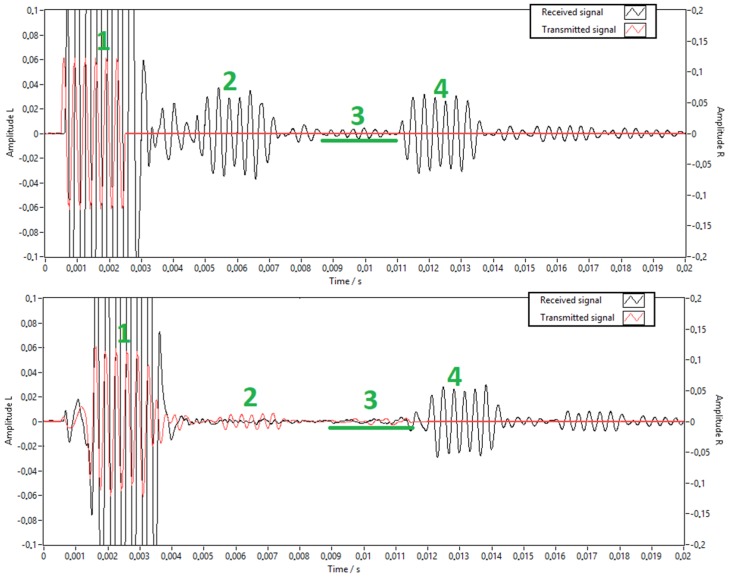
Comparison of returned signals before (top) and after acoustic signal cancellation (ASC) (bottom). The region where signal overlap occurs is shown by the green line. With 1 is marked direct signal from the loudspeaker, with 2 is marked returned signal from change in radius (marked in Figure 6), with 4 is marked signal from the end of the sensing end. With 3 is marked overlapped signal which is first reflected from change in radius and again from the loudspeaker.

One of the limitations of a single tube PAT design is the overlapping of the returned acoustic signals [21] due to reflections of the acoustic waves at the loudspeaker (see Figure 1 and Figure 14) and the finite length of common sound path (*l_c_* is 0.6 m). This combination creates slowly decaying acoustic waves, which cause significant acoustic overlap between the returned signals and limit the sample rate of the PAT (14 Hz). Similar problems also occur with other acoustic time-of-flight applications [22]. Signal overlap depends on the speed of sound in the common sound path, which is influenced by the temperature in the common sound path part of the tube. When tested in a water bath at 70 °C, the maximum difference was 0.1 °C, depending on the depth of the PAT submersion. This is an unwelcome effect because temperature in the sensing end is influenced by the environment temperature of the common sound part of the tube. To minimise the unwanted return signals, a loudspeaker can send sound pulses to cancel the unwanted acoustic reflections.

In addition to eliminating the first reflection, this algorithm also shapes the transmitted signal into a more ideal form. This is beneficial as the loudspeaker that is used has its own frequency response, which is not ideal (the same is also true for the microphone, but the loudspeaker had a larger deviation from the ideal frequency response than the microphone) and can be corrected to compensate for the possible drift in frequency response.

In Figure 14, the signals before and after acoustic signal cancellation (ASC) are shown. The received signal is marked in black and the transmitted signal in red. The excitation signal was a gated sine wave with six cycles. A partial signal overlap can be seen above the green line on the upper figure, the lower figure only contains the remaining attenuated low frequency noise. The scale for the transmitted signal is on the right and the scale for the received signal is on the left. Signal range is from −1 to 1. It can be seen that the received signals from the first reflection are almost completely eliminated (suppressed to one third of the unsuppressed signal) by the additional signals produced by the loudspeaker.

Signal cancellation was performed in the frequency domain by minimising the difference between the received and desired spectrum. Details of this algorithm are explained in [7]. This was done iteratively throughout the entire measurement time. The next signal (spectrum) was determined by:

(13) $$E_{n}=\frac{I\left(\omega \right)}{R_{n-1}\left(\omega \right)}\cdot E_{n-1}\left(\omega \right)\cdot k+\left(1-k\right)\cdot E_{n-1}\left(\omega \right),$$

where *I*(ω) is the spectrum of desired returned signal, *R_n_*_−1_(ω) is the spectrum of the previous returned signal, *E_n_*_−1_(ω) is the previous transmitted signal, and k is the stability parameter, which defines how fast the output signal should change to ensure convergence. The value of the stability parameter *k* primarily depends on the delay between the transmitted and received signals. Five signal packets were processed at a time because of the hardware limitations in this design. For this configuration, the value of *k* = 0.001 ensured reasonably fast convergence and stability.

### Calculating the Optimal Tube Lengths for the Single Tube PAT Design

**Figure 15:**
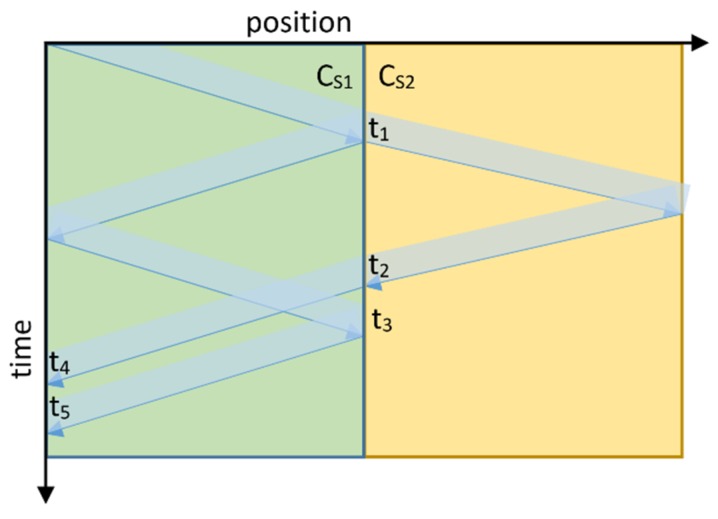
Timing diagram of the acoustic signals in the tubes. Green region marks common sound path and yellow marks sensing end. Blue shadow marks presence of acoustic signal at specific point in time and position.

Another way to reduce or remove acoustic signal overlap is to design a thermometer with optimal tube lengths. The tube length influences parameters such as acoustic SNR, temperature sensitivity, and acoustic signal overlap. A decrease in the length of the sensor tube increases the SNR and reduces acoustic signal overlap, but also reduces temperature sensitivity, which is important for finding the optimal tube lengths. To calculate the optimal length, one needs to choose certain acoustic signals and instrument parameters beforehand. These parameters are the duration of excitation signal, temperature range, and the properties of the gas used. Knowing the values of these parameters in advance is important because it is difficult to alter them later, as they can only be changed in discrete steps. The temperature range and gas properties are used to calculate the minimum and maximum speed of sound in the tubes. Equations (14)–(16) present conditions for the tube lengths where acoustical signals do not overlap.

(14) $$l_{c}\geq \frac{t_{d}c_{s1}}{2}$$

(15) $$l_{s}\geq \frac{t_{d}c_{s2}}{2}$$

(16) $$l_{s}\leq \frac{c_{s2}}{2}\left(\frac{2l_{c}}{c_{s1}}-t_{d}\right)$$

where *l_c_* is the length of the common sound path, *l_s_* the length of the sensing end, *t_d_* is the duration of the acoustic signal, and *c_s_*_1_ and *c_s_*_2_ are the sound velocities in the common to the sound path and the sensing end. From Equations (14) and (15), we can calculate that reflections from end of tubes must not return to their origin during transmission. Equation (16) states that the entire acoustic signal has to return from the sensing end to change radius before the signal part that was reflected from the origin (this can be seen in Figure 15, as *t*_2_ has to be less than *t*_3,_ for *t*_4_ to be less than *t*_5_). In addition to the equations, there are two contradicting requirements: (i) to reduce the length of the common sound path (to reduce physical size of instrument) and (ii) to increase the length of the sensing end (to increase sensitivity of instrument). An optimum can therefore be found when the left-hand side of Equations (14) and (16) is equal to the right hand side. Figure 15 is a timing diagram illustrating why and when a signal overlap occurs. The blue arrow marks the wave front of the acoustic signal and the blue shadow represents the duration of the signal. Green and yellow colours mark regions with a speed of sound *c_s_*_1_ and *c_s_*_2_, respectively.

The acoustic signal starts at the top left corner and arrives at the change in radius (i.e., start of the sensing end) at time *t*_1_, when it splits into two parts. One part of the signal is reflected back to the origin and a second part continues towards the end of sensing end. Both parts of the signal are then reflected back to the change of radius. They arrive back at the change in radius at times *t*_2_ and *t*_3_. Afterwards, they continue towards the origin where they are captured by the microphone at times *t*_4_ and *t*_5_. Signal overlap occurs when the time difference between *t*_4_ and *t*_5_ is less than the duration of the acoustic signal. The difference between *t*_4_ and *t*_5_ is the same as the difference between *t*_2_ and *t*_3_, so to ensure there is no overlap the signal reflected from the sensing end must arrive at the radius change before the part of the signal that was reflected from the origin.

There are other additional reflections beside the first two, however they do not disturb the first two reflections which are used to measure the acoustic time delay. These additional reflections do influence the following time delay measurement, unless there is a pause between measurements. For this PAT design it was measured that a pause of 68 ms or longer is sufficient to remove this influence.

## 6. Measuring Time Delay with a Frequency Modulated Continuous Wave Acoustic Signal (CWFM)

**Figure 16:**
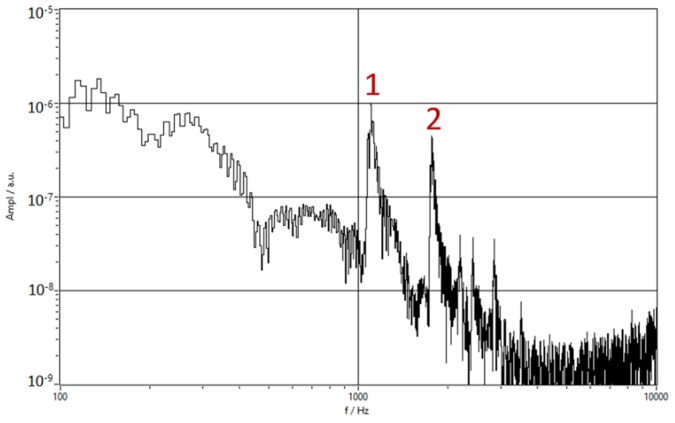
FFT spectrum of the returned signal, the marked peaks were used to calculate speed of sound.

A CWFM signal is used in radar systems to determine the distance from the stationary and moving targets. The same principle could be used to estimate the time delay in the PAT. The transmitter repeatedly sends signals with modulated frequency [23,24]. CWFM radars are mainly used in combination with electromagnetic (EM) waves for measuring the distance to stationary objects. The common use of CWFM radars is to measure unknown distance by using the fact that the speed of the EM waves is known. The PAT uses the CWFM technique in reverse, the distance is known, and the speed of sound is measured. While using this with acoustic waves is much more limited due to the higher attenuation compared with EM waves, it is still possible to get meaningful speed of sound measurements. In Figure 16 the fast Fourier transform (FFT) spectrum of the returned signal is shown. Two peaks are marked, which represent reflection from the start of the sensing end (1) and the reflection from end of sensing end (2). Other peaks in spectrum represent signals with multiple reflections on their path.

The speed of sound can be calculated as:

(17) $$c_{s}=\frac{2l_{s}}{t_{r}},$$

where *t_r_* is:

(18) $$t_{r}=\frac{\Delta f_{echo}}{k_{r}\;},$$

where ∆*f_echo_* is the difference in frequency of the first and second reflections in the sensing end, as shown in Figure 16 and *k_r_* is:

(19) $$k_{r}=\frac{\Delta f}{\Delta t}\;,$$

where ∆*f* is the bandwidth of the chirp signal and ∆*t* is the duration of one chirp signal.

**Figure 17:**
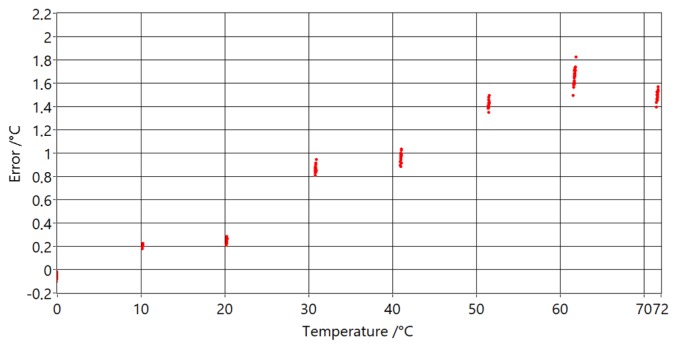
Temperature error versus reference temperature readings with speed of sound calculated by the Frequency Modulated Continuous Wave Acoustic Signal (CWFM) technique with separated microphone and loudspeaker.

In Figure 17 the preliminary results of the PAT with the CWFM technique are shown. As can be seen, the PAT temperature measurements are closely related to the reference temperature. The main problem is the dispersion of sound in the tubes.

The CWFM principle was successfully demonstrated with a separate microphone and loudspeaker, using the same PAT hardware as described in Section 7.

**Figure 18:**
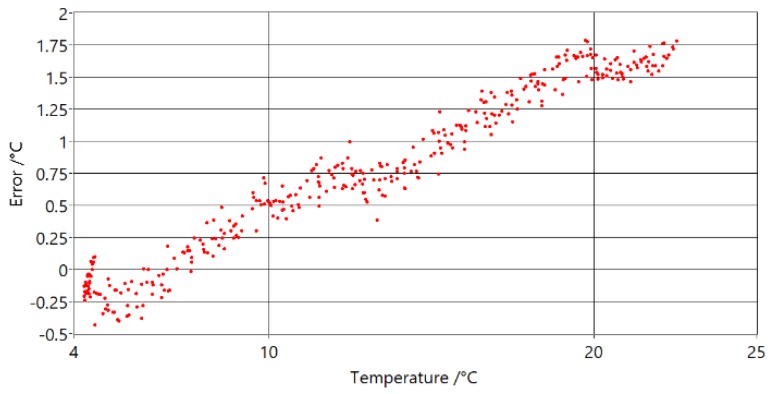
Temperature error versus reference temperature readings for single transducer CWFM.

The single transducer CWFM principle was also successfully demonstrated, although with lower sensitivity, due to the limited voltage output of the hardware. The results from this limited test are shown in Figure 18. This test was performed with air as the measurement gas at atmospheric pressure.

With the single transducer principle, the PAT design is substantially simplified.

## 7. Experimental Setup

After the instrument was vacuumed and filled with argon, it was left at the ice point to measure its drift. The first test at ice point was used for calibration, to determine the length between reflective surfaces. Once the acoustic delay at 0 °C is known, it is straightforward to calculate length of the sensing end. The evaluation consisted of a number of stable temperatures from 0 to 70 °C in a water bath and at the ice point. The uncertainty (homogeneity and stability) of the used temperature bath was estimated at below 5 mK. For a reference thermometer, we used standard platinum resistance thermometers [25] (SPRT) with an uncertainty below 1 mK, calibrated at fixed points. The PAT measured the temperature 14 times per second and the reference thermometer once every 10 s. The PAT was mounted vertically with the last 10 cm of common sound path above water or ice level.

**Figure 19:**
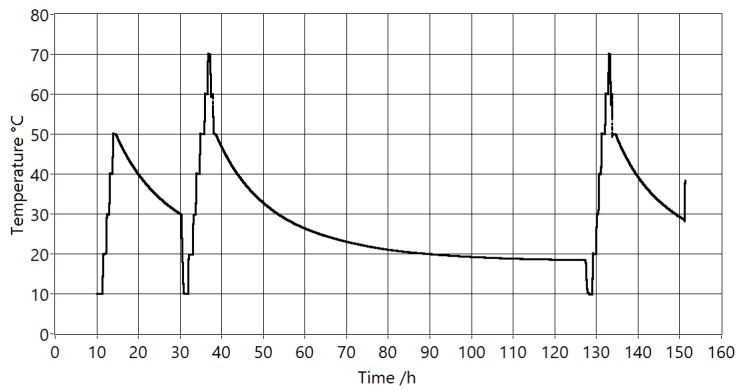
Temperature profile of the reference thermometer measurements during the evaluation.

The temperature profile of the reference thermometer during the evaluation is shown in Figure 19. It consists of 22 stable temperature points at 10, 20, 30, 40, 50, 60, and 70 °C with a minimum dwell time of 30 min. Each temperature point was repeated at least twice. This part of the evaluation spanned five days.

## 8. Results and Discussion

For the evaluation of the models of the speed of sound in the tubes and the error analysis we used the properties of argon that are listed in the NIST WebBook [26]; the other values were *f* = 3 kHz, *l_c_* = 0.6 m, *l_s_* = 0.9 m, and *R* = 2.5 mm. These values also correspond to the PAT prototype with ASC.

The results of the model comparison suggest that the propagation of sound inside tubes with a higher-pressure medium appears to be more like that in the free field. Increasing the pressure also benefits the temperature measurement, as it increases the overall SNR of the system. Another important result is that, for high temperature measurements, it is enough to consider the simplified model of the propagation constant. For measurements at lower temperatures or very precise measurements, it is necessary to use the complete model for the propagation constant.

### 8.1. Results of the PAT with Acoustic Signal Cancellation

**Figure 20:**
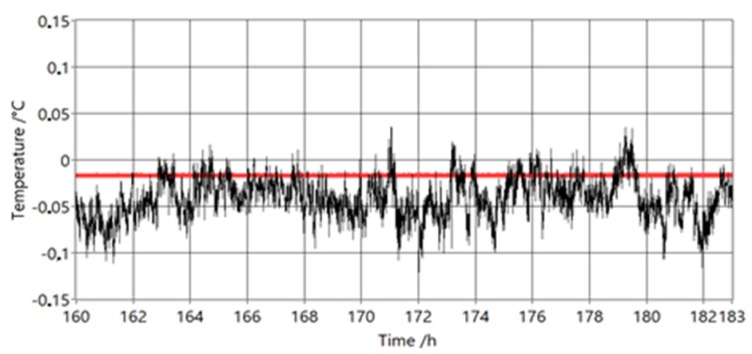
Temperature readings at calibration at ice point (last day). The red colour represents the reference thermometer and the black colour represents the raw un-averaged PAT measurements.

Initial calibration was performed at ice point. Also, this measurement was used to determine the possible drift of the PAT due to the effects of the mixing of the gases inside the PAT. In Figure 20, we can see a measurement excerpt, which is also used for calibration of the instrument.

The reference thermometer measurements are shown in red and the PAT measurements are shown in black. There was no noticeable drift after one week of measurements.

**Figure 21:**
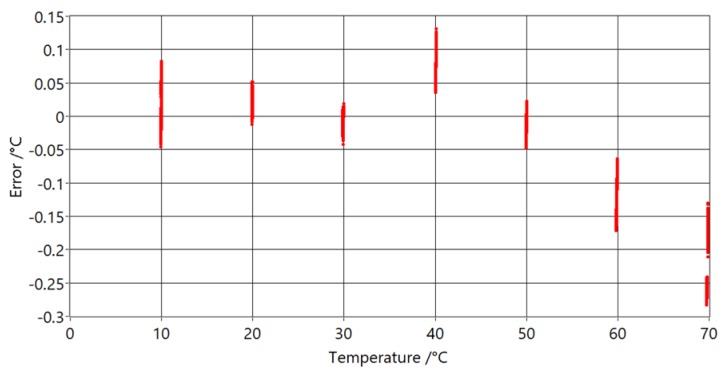
Temperature error versus reference temperature readings with used ASC algorithm.

Differences between the PAT and reference thermometer differences at temperatures are shown in Figure 21.

It can be seen that measurements below 40 °C are a good match to the model used (differences are less than 0.1 °C), but at higher temperatures they start to deviate from it. This is due to acoustic signal overlap, when signals from different reflection points arrive at the microphone at same time. The most probable reason for why the deviations start at higher temperatures (40 °C and increase with increasing temperature, is that water from the temperature bath evaporates and heats the common sound path which is above the water level. The other significant factor for the increase of temperature in the common sound path is the thermal conductivity of the tube.

The final evaluation was performed one month after initial calibration to determine the effects of gas replacement. The inside of the PAT was vacuumed and filled with argon from the same gas canister. The PAT did not show any measurable difference compared with previous measurements at ice point.

The standard deviations of temperature for chirps and single frequency tone are presented in Table 2. In parentheses are the standard deviations of the time delay measurements. Measurements were done at stable temperature inside the bath with an uncertainty of 5 mK, which includes bath stability, homogeneity, and the uncertainty of the SPRT that was used. The standard deviation was calculated using 20 temperature (time delay) measurements. It can be seen that temperature measurements using single frequency tone signals have a smaller standard deviation, and as such are better for measuring time delay in combination with chirps than using chirps alone. The standard deviations of the time delay measurements show that the precise time delays of the acoustic signal measurements are required. This is more problematic at higher temperatures because of quadratic relationship between temperature and time delay (speed of sound).

During the tests, a problem emerged due to vibration and acoustic noise from the environment. While external sound could be detected with the microphone inside PAT, it did not increase the noise of the temperature measurements. On the other hand, vibrations can be more problematic. For example, vibrations from the compressor and motor in the bath working at full power, increased the standard deviation of temperature measurements at 30 °C to 20 mK.

### 8.2. Results of the PAT with Optimal Waveguide Lengths

Properties for this prototype are: *f* = 6 kHz, *l_c_* = 0.9 m, *l_s_* = 0.45 m, and *R* = 2.5 mm. The duration of the gated sinewave is 1 ms followed by 70 ms of silence.

**Figure 22:**
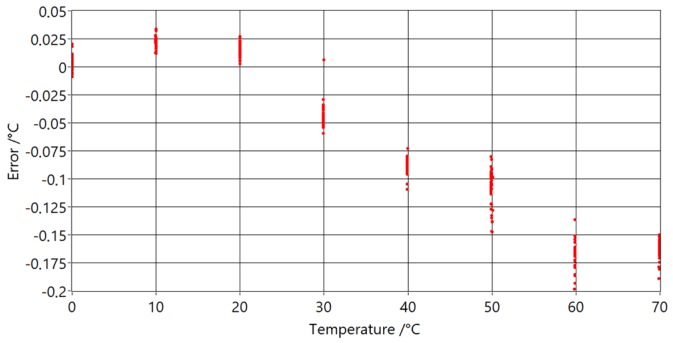
Temperature error versus reference temperature readings for PAT with optimal waveguide lengths.

Similar to the prototype with ASC, this prototype was calibrated at ice point and then moved to a water bath for comparison with the reference thermometer. In Figure 22, the differences between the PAT and reference thermometer difference at temperatures are shown.

Compared with measurements of the PAT with ASC, shown in Figure 21, it can be seen that these measurements have smaller standard deviations and smaller maximum deviations from model. The influence of the heat transfer of the tube and gas was tested by changing the immersion depth of the sensing part at the extremes of the temperature range. The depth of the sensing part was changed from 40 cm (maximum depth) to 20 cm, with no significant influence on the measurements.

### 8.3. Results of the Monte Carlo Simulation

The result of the MC simulation of the complete model of speed of sound includes combined uncertainty from all parameters, and partial contributions from each parameter to find which parameter contributes the most uncertainty. Table 3 shows the parameters, the sensitivity coefficients, and their contribution to type B uncertainty at 0 °C and 400 kPa.

It is clear that the largest influential contributor is the uncertainty of the speed of sound. Other parameters contribute to the uncertainty but in a much smaller way than the speed of sound. This means that the most important factor in reducing PAT uncertainty is the use of highly accurate speed of sound data. Current measurements of sound delay [18] have similar uncertainty contributions as other parameters (other than speed of sound), which means that the proposed algorithm for measuring the speed of sound is comparable to other sources of uncertainties. Error analysis showed that the type B uncertainty of the PAT with a single tube was 65 mK.

**Figure 23:**
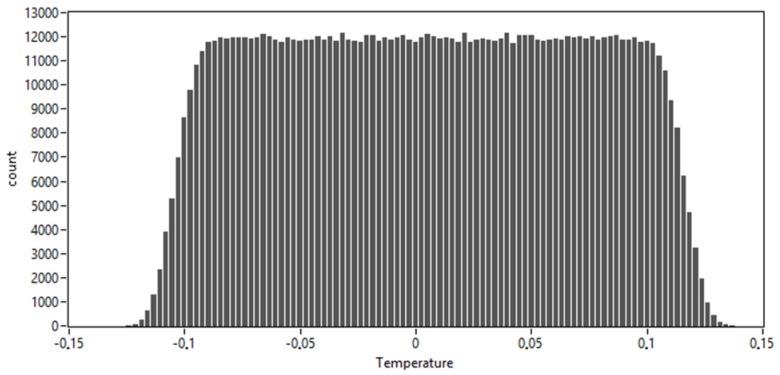
Histogram from the MC simulation at 0 °C.

Figure 23 shows a histogram from a single simulation run at 0 °C and 400 kPa. The data from the simulation are gathered in 100 bins. The shortest 95% coverage interval is from −115 to 101 mK.

While the ASC algorithm reduced the acoustic signal to only one third of the original signal intensity, there was still too large an error in the temperature measurement. Additionally, it increased the measurement uncertainty of the sound delay measurements by introducing low frequency artefacts in the acoustic signal. As ASC could not solve the acoustic signal overlap problem completely, we decided to use another technique without ASC. The core of this technique is the optimal selection of tube length, which mostly depends on the nature of the measurement gas inside the tubes and the temperature range.

## 9. Conclusions

When using a PAT of any design, it is important to consider the measured physical parameters of the real gases used. Relying on models of ideal gas will not ensure a good agreement with the model, especially at lower temperatures (e.g., −100 °C) where the speed of sound in real gas significantly deviates from the speed of sound in ideal gas. We showed that a simplified model was still valid despite the assumptions that were violated. Error analysis showed that type B uncertainty of the PAT with a single tube was 65 mK. While ASC was useful in masking the overlapped return signals, it was unable to completely eliminate the effects of overlapping signals on the temperature measurement. Another problem was the increased acoustic noise while masking the acoustic signals. Another possible solution for minimising the signal overlap in a selected temperature range is a change in the PAT tube length. Despite the fact that the sound delay measurement makes a small contribution to the overall temperature uncertainty, it is possible to decrease it even further using a higher gas pressure inside the thermometer tube. This should be useful in environments with high acoustic and vibration noise because the acoustic SNR of the thermometer increases with increased gas pressure.

## Figures and Tables

**Table 1:** Parameters of Equation (3) and the associated uncertainties.

Parameters With Rectangular Probability Distribution		
	Expectation	Semi-Width
Heat capacity c_p_	21.03 J∙mol^−1^∙K^−1^	0.06 J∙mol^−1^∙K^−1^
Dynamic viscosity	21.1 µPa∙s	0.4 µPa∙s
Thermal conductivity	16.6 mW∙m^−1^∙K^−1^	0.3 mW∙m^−1^∙K^−1^
Speed of sound	307.979 m∙s^−1^	0.061 m∙s^−1^
Heat capacity c_v_	12.51 J∙mol^−1^∙K^−1^	0.03 J∙mol^−1^∙K^−1^
Thermal linear expansion	−0.03%	0.15%
Parameters with normal probability distribution		
	Expectation	STD
Sound delay	0.006163 s	22 ns
Pressure	400000 Pa	3 Pa

**Table 2:** Standard deviation of temperature measurements of chirps and single frequency tone at different temperatures. The standard deviations of measured time delays are shown in the parentheses.

Temperature	Standard Deviation	
	Gated sinewave	Chirps
10 °C	5.6 mK (60 ns)	9.3 mK (98 ns)
30 °C	8.7 mK (83 ns)	14 mK (133 ns)
70 °C	7.9 mK (62 ns)	8.5 mK (67 ns)

**Table 3:** Results of Monte Carlo (MC) simulation.

Parameter	Sensitivity Coefficient	Uncertainty Contribution
Heat capacity c_p_	0.026 K/J∙mol^−1^∙K^−1^	0.9 mK
Dynamic viscosity	0.005 K/µPa∙s	1.1 mK
Thermal conductivity	0.006 K/mW∙m^−1^∙K^−1^	1.0 mK
Speed of sound	1.648 K/m∙s^−1^	58.1 mK
Heat capacity c_v_	0.058 K/J∙mol^−1^∙K^−1^	1.0 mK
Thermal linear expansion	0.013 K/%	1.1 mK
Sound delay	0.045 K/µs	1.0 mK
Pressure	0.3 mK/Pa	0.9 mK
